# Deep sequencing of small TAR-derived RNAs in HIV-1 producing cells

**DOI:** 10.1186/1742-4690-8-S2-P31

**Published:** 2011-10-03

**Authors:** Alex Harwig, Ben Berkhout, Atze T Das

**Affiliations:** 1 Laboratory of Experimental Virology, Department of Medical Microbiology, Center for Infection and Immunity Amsterdam (CINIMA), Academic Medical Center of the University of Amsterdam, The Netherlands

## Background

HIV-1 encodes the same stable stem-loop structure at both the 5' and 3' end of all viral RNA transcripts. This TAR hairpin is essential for HIV-1 replication because it binds the viral Tat transcriptional activator protein. Several groups have shown that intracellular processing of TAR RNA by the Dicer endonuclease of the RNA interference (RNAi) pathway results in the production of a miRNA that can affect cellular gene expression via this RNAi pathway [1-4], but others were not able to detect such small TAR-derived RNAs [5]. We here used the sensitive SOLiD ultra-deep sequencing method to detect small RNAs corresponding to TAR in HIV-1 expressing cells.

## Materials and methods

We transfected 293T cells with plasmids encoding the complete genome of the HIV-1 LAI strain or the HIV-rtTA variant. In HIV-rtTA, the Tat-TAR transcription mechanism has been replaced by the doxycycline-inducible Tet-On system. This modification allowed the introduction of mutations in TAR and Tat. Intracellular RNA was isolated at 2 days post-transfection and analyzed by SOLiD sequencing. Expression of TAR-derived small RNAs was also investigated by Northern blot analysis of RNA isolated from cells.

## Results

SOLiD analysis of RNA from HIV-1 LAI and HIV-rtTA expressing cells resulted in approximately 17 million short RNA sequences (14-35 nt) per sample, with approximately 55 thousand sequences of viral origin. For the HIV-1 LAI sample, 1% of the viral sequences were derived from the 3'side of the TAR stem. This set of TAR-derived small RNAs overlapped considerably, but varied in size (14-21 nt). In the HIV-rtTA samples, a significantly larger fraction (50-70%) of the sequences were TAR-derived. Mutational inactivation of the Tat gene did not affect the production of the small TAR-derived RNAs. Northern blot analysis of intracellular RNA from HIV-1 LAI expressing cells not only confirmed the presence of the small TAR-derived RNAs, but also revealed the presence of short TAR-containing transcripts (60-150 nt). The level of these short viral transcripts was much increased in HIV-rtTA versus LAI, and we observed a concomitant increase in the TAR-derived small RNAs, in agreement with the SOLiD analysis. We speculate that the small TAR RNAs are produced by processing of short HIV-1 transcripts that are made by non-processive transcription from both LTR promoters. Analysis of the processing products of different TAR hairpin mutants allowed us to probe the specificity of the RNA processing process.

## Conclusions

Transcription of the HIV-1 genome results in the production of short TAR-containing transcripts that seem to be the precursor for the abundant small TAR-derived RNAs. The initial processing results with TAR hairpin mutants does not support a role for Dicer in this process, but further analysis is required to study the generation of TAR miRNAs.
